# The Protective Effects of Ultraviolet A1 Irradiation on Spontaneous Lupus Erythematosus-Like Skin Lesions in MRL/lpr Mice

**DOI:** 10.1155/2009/673952

**Published:** 2009-04-26

**Authors:** Naoya Mikita, Nobuo Kanazawa, Takashi Yoshimasu, Takaharu Ikeda, Hong-jin Li, Yuki Yamamoto, Fukumi Furukawa

**Affiliations:** Department of Dermatology, Wakayama Medical University, 811-1 Kimiidera, Wakayama 641-0012, Japan

## Abstract

We investigated the effects of ultraviolet A1 (UVA1) irradiation on spontaneous lupus erythematosus- (LE-) like skin lesions of MRL/lpr mice, using a disease prevention model. UVA1 irradiation significantly inhibited the development of LE-like skin lesions, without obvious changes of the disease including renal disease and serum antinuclear antibody levels. Besides the massive infiltration of mast cells in the LE-like skin lesions, in the nonlesional skins, more mast cells infiltrated in the UVA1-irradiated group compared with the nonirradiated group. Although apoptotic cells were remarkably seen in the dermis of UVA1-irradiated mice, those cells were hardly detectable in the dermis of the nonirradiated mice without skin lesions. Further analysis showed that some of those apoptotic cells were mast cells. Thus, UVA1 might exert its effects, at least in part, through the induction of the apoptosis of pathogenic mast cells. Our results supported the clinical efficacy of UVA1 irradiation for skin lesions of lupus patients.

## 1. Introduction

Collagen disease, one of the representative autoimmune diseases, is often accompanied by photosensitivity. In particular, photosensitivity is included in its diagnostic criteria of systemic lupus erythematosus (SLE) [1, 2], and it has been reported that, in Japan, about 60% of SLE patients showed photosensitivity [3, 4]. In addition, exposure to sunlight is a well-known risk factor that induces or exacerbates not only skin lesions but also systemic symptoms for SLE patients. To investigate the effects of sunlight on SLE, some studies using MRL/lpr mice, one of the major lupus-prone mice, have been performed. MRL/lpr mice are characterized by dysregulated apoptosis due to defective signaling through Fas antigen [5] and by autoimmune symptoms including fetal nephritis, autoantibody production, and lymphoadenopathy. Furthermore, some of MRL/lpr mice spontaneously develop LE-like skin lesions with alopecia and scab formation on their upper back region [6–8]. In cultured cells from MRL/lpr mice, the cytotoxicity of ultraviolet (UV) irradiation was significantly elevated [9]. In line with this, UVB exposure significantly exaggerated the development of LE-like skin lesions in MRL/lpr mice [10]. Moreover, UVA could also exacerbate the skin lesions in SLE patients [11]. On the contrary, several recent studies demonstrated that UVA1 irradiation had therapeutic effects for SLE patients [12–15].

 Ultraviolet can be divided into three different parts based on the wavelength as follows: UVA (320–400 nm), UVB (290–320 nm), and UVC (200–290 nm). Moreover, UVA is made up of two parts: UVA1 (340–400 nm) and UVA2 (320–340 nm). UVC is absorbed by the ozone layer and does not reach the ground, whereas UVA and UVB have different various biological effects. For example, UVA and UVB induce suntan without an erythema response and suntan with sunburn, respectively [16, 17]. Although the biological effects of UVA2 are similar to the effects of UVB, only UVA1 possesses the particular characteristic biological functions. In the previous studies with MRL/lpr mice [9, 10], the biological effects of UVB but not UVA1 were mainly investigated. McGrath Jr. et al. reported the systemic effects of UVA, not UVA1, on other SLE model mice (NeaZealand black × NeaZealand white), F1 hybrid mice harboring no skin lesions [18]. However, to the best of our knowledge, there have been no studies examining the UVA1 effects on the LE-like skin lesions of SLE model mice, and the biological effects of UVA1 on SLE still remain investigated. In the present study, we investigated the effects of UVA1 irradiation on MRL/lpr mice, particularly on their LE-like skin lesions, using a disease prevention model. Simultaneously, we also studied the dermal infiltration of mast cells, histamine receptor (HR) expression [19] and the mRNA expression of cytokines [20], which were previously examined in the spontaneous LE-like skin lesions in MRL/lpr mice [19, 20].

## 2. Materials and Methods

### 2.1. Antibodies (Abs)

In the present study, the following Abs were used: rabbit antimouse histamine 1 receptor (H1R) polyclonal Ab (pAb), goat antimouse H2R pAb, goat antimouse H3R pAb (Santa Cruz Biotechnology, Inc., Santa Cruz, Calif, USA), and rat antimouse IL-10 monoclonal Ab (mAb) (BD Biosciences, San Jose, Calif, USA).

### 2.2. Mice

MRL/lpr mice, aged 4–6 weeks at the start of each experiment, were purchased from Japan SLC Inc. (Hamamatsu, Japan) and housed individually in cages under the specific pathogen free conditions. All animal experiments were approved by the Committee on Animal Care and Use at Wakayama Medical University.

### 2.3. UVA1 Sources and Irradiation of Mice

The UVA1 source was a TLK/40W/10R/UVA PHILIPS lamp (PHILIPS, Amsterdam, The Netherlands). This lamp emitted long wave UV light ranging from 350–400 nm (within the UVA1 range (320–400 nm)) with a peak at 365 nm. The UVA1 irradiation equipment consisted of four of these lamps (Yayoi Co., Ltd., Tokyo, Japan).

The MRL/lpr mice were divided into the following three groups: (1) nonirradiation group (*n* = 22), (2) UVA1 irradiation with 5 J/cm^2^ (*n* = 18), and (3) UVA1 irradiation with 10 J/cm^2^ (*n* = 18). The doses of 5 and 10 J/cm^2^ have been chosen by the following reasons: first, effective doses of UVA1 treatment for human SLE patients were reportedly low, such as 6 and 12 J/cm^2^ [12–14]; second, it took about 60 minutes to irradiate 10 J/cm^2^ of UVA1 using our equipment, and therefore the irradiation over 10 J/cm^2^ seemed quite stressful for mice. Under the deep anesthesia, all of mice were shaved at the upper dorsal skin twice a week, because the spontaneous LE-like skin lesions would appear on this site at older ages. Mice were irradiated UVA1 (5 J/cm^2^ or 10 J/cm^2^) five times a week for 4 months. The nonirradiated mice were left untreated after shaving. LE-like skin lesions of mice were macroscopically evaluated. The mortality was also estimated during experimental period.

### 2.4. Proteinuria

At 4 months after the first UVA1 irradiation, urine samples were collected, and their protein contents were quantitatively tested in order to monitor the onset and degree of renal disease using ALBUSTIX urine test paper (Miles-Sankyo Co., Ltd, Tokyo, Japan). Proteinuria was evaluated as negative (0–30 mg/dL), 1 + (30–100 mg/dL), 2 + (100–300 mg/dL), and 3 + (300–1000 mg/dL).

### 2.5. Measurements of Antinuclear Antibody (ANA) in Sera

Serum samples, collected at 4 months after the first irradiation, were subjected to ANA analysis by a commercial kit (Fluoro HEPANA test, MBL Co., Ltd, Nagoya, Japan), as described previously [21]. 

### 2.6. Light Microscopic Observation of the Skin

At 4 months after the first irradiation, mice were euthanized by the cervical dislocation, and skin specimens were taken from the upper back regions of all mice. The specimens were fixed in 4% formaldehyde buffered with PBS (pH 7.2), embedded in paraffin, and stained with hematoxylin and eosin (HE). Toluidine blue staining was also performed to assess the infiltration of mast cells. For the evaluation of dermal infiltrating mast cells, 5 microscopic fields were randomly selected, and the number of mast cells was counted. The degree of mast cells infiltration was expressed as the average number of the mast cells in the 5 microscopic fields (magnification, ×100). All measurements were performed without prior knowledge of the experimental procedures.

### 2.7. Light Microscopic Observation of the Kidney

Kidney specimens taken from all MRL/lpr mice were subjected to HE and periodic acid-Schiff (PAS) staining. Then, the degree of glomerulonephritis was evaluated according to previously described methods [22] with a slight modification. All measurements were performed without prior knowledge of the experimental procedures.

### 2.8. Immunohistochemical Study

Skin specimens were frozen immediately in Tissue-Tek O.C.T. Compound (Sakura Finetechnical Co., Ltd, Tokyo, Japan) to be stored at −80°C until the use. Cryosections (6 *μ*m thick) were prepared using Cryostat CM1900 (Leica Microsystems, Heidelberg, Germany), and the sections were reacted with anti-H1R pAbs, anti-H2R pAbs, or anti-H3R pAbs. After the incubation of biotinylated secondary Abs, immune complexes were visualized using VECTASTAIN Elite ABC Kits (Vector Laboratories, Burlingame, Calif, USA), according to the manufacturer's instructions. 

### 2.9. Immunofluorescence Study

To investigate the immunoglobulin deposits in the skin, cryosections were immunostained with Alexa Fluor 488-conjugated antimouse IgG (Invitrogen, Eugene, Ore, USA). In another series, to evaluate the IL-10 production by mast cells, cryosections were double-immunostained with rat antimouse IL-10 mAb and avidin-Texas Red (BD Biosciences, San Jose, Calif, USA). After the incubation with Alexa Flour 488-conjugated antirat IgG (Invitrogen) as secondary Ab, the stained sections were observed with Radiance 2100 Confocal Microscope System (Bio-Rad Laboratories, Hercules, Calif, USA).

### 2.10. Terminal Deoxynucleotidyl Transferase- (TdT-) Mediated dUTP Nick End Labeling (TUNEL)

Apoptotic cells were detected by the use of In Situ Cell Death Detection Kit, Fluorescein (Roche Diagnostics KK, Tokyo, Japan), followed by nuclear labeling with Hoechst 33342 (Invitrogen). Moreover, some sections were incubated with avidin-Texas Red prior to in situ TUNEL.

### 2.11. Reverse Transcription-Polymerase Chain Reaction (RT-PCR) Analysis

The levels of mRNA expression of TNF-*α*, IFN-*γ*, IL-4, IL-10, TGF-*β*, H1R, H2R, and H3R were determined by RT-PCR analyses, as described previously [19–21]. Briefly, the total RNAs were extracted from dorsal skin specimens with Sepazol-RNA I (Nacalai Tesque, Inc., Kyoto, Japan) and reverse transcribed using a SuperScript III First-Strand cDNA Synthesis System (Invitrogen) according to the manufacturer's protocol. The PCR was performed using Ex Taq (TAKARA BIO INC., Otsu, Japan) and Applied Biosystems 2720 Thermal Cycler (Applied Biosystems, Foster City, Calif, USA). The PCR products were electrophoresed on a 2% agarose gel and visualized by ethidium bromide staining.

### 2.12. Statistical Analysis

For comparisons on the frequency of LE-like skin lesions and the mortality, Fisher's exact probability test was used. For comparisons on the mast cells recruitment and proteinuria, Student's *t*-test was employed. *P*-values less than .05 were regarded as statistically significant.

## 3. Results and Discussion

### 3.1. Protective Effects of the UVA1 Irradiation on the Development of LE-Like Skin Lesions in MRL/lpr Mice

**Figure 1:**
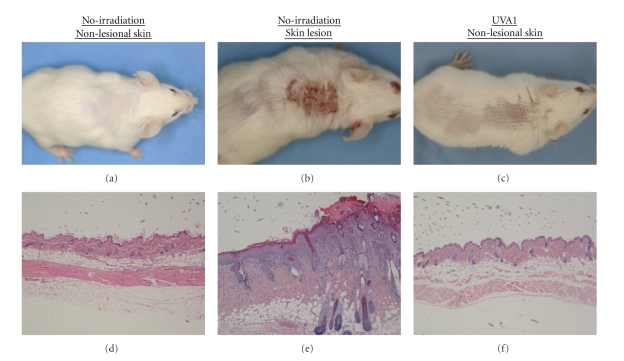
Macroscopic and histopathological findings of the skin representative of MRL/lpr mice. (a), (d): nonlesional skin of nonirradiated mice. (b), (e): spontaneous LE-like skin lesion of nonirradiated mice. (c), (f): nonlesional skin of UVA1-irradiated (10 J/cm^2^) mice. As shown in Table 1, some of the nonirradiated MRL/lpr mice developed LE-like skin lesions with alopecia and scab formation (b). In the spontaneous LE-like skin lesions, histopathologically, acanthosis with hyperkeratosis, liquefaction, and mononuclear cell infiltrations into the dermis were noted (e). On the other hand, none of the UVA1-irradiated mice showed LE-like skin lesions (c). Most of the nonlesional mice did not show any histological changes (d), (f). (d), (e), (f): HE stain, original magnification, ×60.

 At the end point of the experiments, 6 (27.3%) out of 22 MRL/lpr mice without UVA1 irradiation apparently developed the macroscopic spontaneous LE-like skin lesions with hair loss and scab formation on their upper back region (Table 1, Figure 1(b)). On the other hand, macroscopic skin lesions were never seen in all UVA1-irradiated (5 J/cm^2^ or 10 J/cm^2^) mice (Table 1, Figure 1(c)). As reported previously [6–8], nonirradiated MRL/lpr mice with macroscopic skin lesions showed characteristic histopathological alterations such as acanthosis with hyperkeratosis, liquefaction, mononuclear cell infiltration into the dermis, and slight extravasation of erythrocytes in the upper dermis (Figure 1(e)). Moreover, even 4 nonirradiated mice without macroscopic skin lesions showed moderate histopathological changes mentioned above, while 5 mice in the UVA1 irradiated groups (5 J/cm^2^: 2 mice and 10 J/cm^2^: 3 mice) showed only LE-like histopathological changes. With respect to the development of LE-like skin lesions, there was a significant difference between the nonirradiated and all UVA1-irradiated groups, and between the nonirradiated and UVA1-irradiated (only 5 J/cm^2^) groups (Table 1, *P* < .05). These observations implied that UVA1 irradiation significantly protected mice against the development of spontaneous LE-like skin lesions.

### 3.2. Effects of UVA1 Irradiation on the Mortality, Renal Disease, and Serum ANA Levels

**Figure 2:**
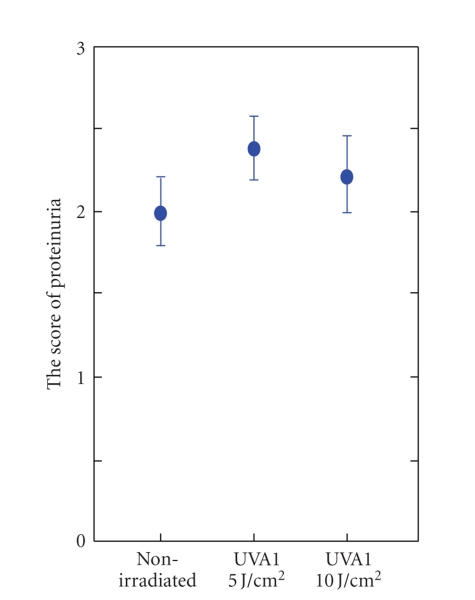
Degree of proteinuria in MRL/lpr mice of the three groups. There were no significant differences among the three groups. UVA1 irradiation had no effect on the degree of proteinuria. The data represent means ± 1 SD.

To assess the systemic effects of UVA1 irradiation on MRL/lpr mice, we evaluated the mortality, the development of renal disease, and serum ANA levels. The mortality of UVA1-irradiated MRL/lpr mice, especially the 10 J/cm^2^ irradiated group, tended to be lower than that of the nonirradiated mice, without statistical significance (Table 2). MRL/lpr mice almost always showed renal disease as evidenced by proteinuria, which was similar to the human lupus nephritis with SLE. UVA1 irradiation had no influence on proteinuria (Figure 2). Furthermore, there was no significant difference in renal histopathological alterations among these three groups (data not shown). Similarly, there was no significant difference in the serum ANA levels and immunoglobulin deposits in skin among three groups (data not shown). These data suggested that, in our experimental procedures, the effects of UVA1 irradiation on systemic symptoms of MRL/lpr mice were not significant. In comparison, some studies on human SLE patients have reported that UVA1 therapy improved the systemic symptoms and parameters [12–15]. Considering the tendency of improving mortality by UVA1 irradiation, further studies of other parameters reflecting systemic immune functions might show some systemic effects of UVA1 in our system.

### 3.3.Effects of UVA1 Irradiation on Dermal Infiltration of Mast Cells

**Figure 3:**
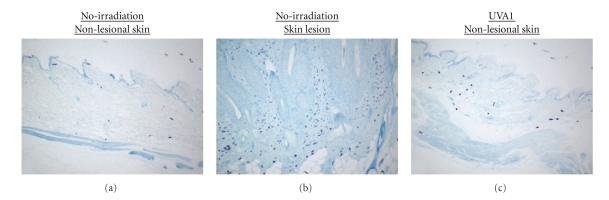
Dermal infiltration of mast cells in the lesional and nonlesional skin representative MRL/lpr mice. (a): nonlesional skin of non-irradiated mice. (b): spontaneous LE-like skin lesion of nonirradiated mice. (c): nonlesional skin of UVA1-irradiated (10 J/cm^2^) mice. In the LE-like skin lesions, the most remarkable infiltration of mast cells into dermis was detected (b). There was more number of mast cells infiltrating into the dermis of the UVA1-irradiated (10 J/cm^2^) mice (c), as compared to the nonirradiated mice (a). Toluidine blue stain, original magnification, ×100.

**Figure 4:**
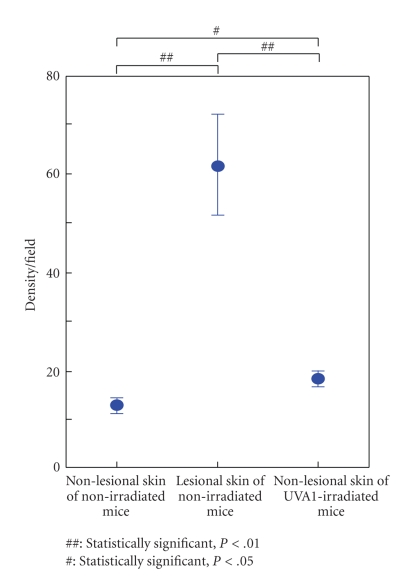
Density of dermal infiltrating mast cells in the skin of MRL/lpr mice. In the LE-like skin lesions of MRL/lpr mice, the density of dermal infiltrating mast cells was the highest as compared with the nonlesional skin of the other mice (*P* < .01). Furthermore, in the nonlesional skins, there was a significant difference between UVA1-irradiated (10 J/cm^2^) and nonirradiated mice (*P* < .05). There was no significant difference between UVA1 5 and 10 J/cm^2^groups. The data represent means ± 1 SD from 5 samples of each group.

**Figure 5:**
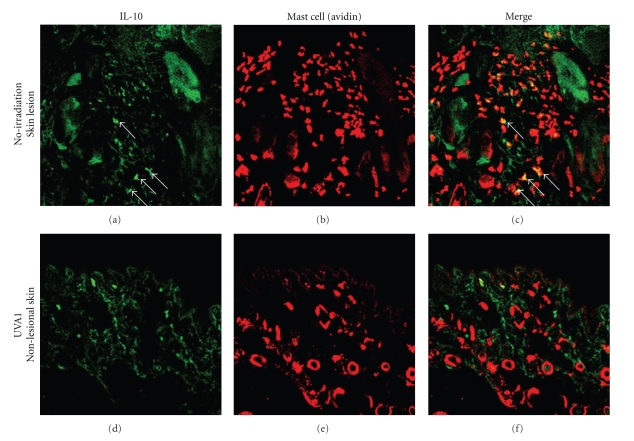
Analysis of the production of IL-10 by mast cells in MRL/lpr mice. (a), (b), (c): spontaneous LE-like skin lesion of nonirradiated mice. (d), (e), (f): nonlesional skin of UVA1-irradiated (10 J/cm^2^) mice. Mast cells are labeled with avidin-Texas Red in red, while IL-10 is immunostained with anti-IL10 mAb and Alexa Fluor 488-conjugated secondary Ab in green. In the LE-like skin lesions, some double immunopositive IL-10-producing mast cells were seen (yellow in (c), indicated by arrows). In contrast, in the nonlesional skin of UVA1-irradiated (10 J/cm^2^) mice, such cells were not detected (f). (a), (d): immunostain for IL-10, (b), (e): immunostain for mast cells, (c), (f): merged images, original magnification, ×200.

In consistent with the previous study [19], we could detect massive infiltration of mast cells in the LE-like skin lesions of MRL/lpr mice, compared with nonlesional skins in both nonirradiated and UVA1-irradiated mice. (Figures 3(b), 4). Furthermore, in nonlesional skins, mast cell recruitment was more evident in UVA1-irradiated mice than in nonirradiated ones (Figures 3(a), 3(c), 4). These observations implied that mast cells might play a role in the protective effects of UVA1 irradiation as well as in the development of spontaneous LE-like skin lesions. Recently, it has been reported that mast cells have both positive and negative regulatory functions for immunity [23]. Mast cells produce IL-10, eventually playing a negative role in the immunomodulatory mechanisms. Actually, UVB irradiation can induce IL-10 production in mast cells [24]. When we examined the IL-10 localization in the skin samples by immunofluorescence analyses, IL-10-positive signals could be detected in the cytoplasm of mast cells at the LE-like skin lesions of nonirradiated mice (Figure 5(c), indicated with arrows). Unexpectedly, mast cells recruited on the skin of UVA1-irradiated mice never showed IL-10-positive signals (Figure 5(f)). Thus, these observations implied at least that UVA1 irradiation could not induce IL-10 production in dermal mast cells in the present study.

### 3.4. Effects of UVA1 Irradiation on the mRNA Expression of Cytokines and the Immunochemical Examination and mRNA Expression of Histamine Receptors

Several lines of accumulating evidence demonstrated that histamine, a major inflammatory mediator mainly derived from mast cells, was closely related with the development of LE-like skin lesions of MRL/lpr mice through HRs [19, 25]. Tachibana et al. suggested that the impairment of histamine metabolism in MRL/lpr mice would contribute to the development of LE-like skin lesions [25]. Actually, the gene expression of H2R was significantly enhanced in the LE-like skin lesions of MRL/lpr mice, and H2R-expressing mononuclear cells could be observed immunohistochemically [19]. The diverse biological effects of histamine are mediated through four different histamine receptors, H1R, H2R, H3R, and H4R [26, 27]. We also examined the expression of the histamine receptors in both the LE-like skin lesion and nonlesional skins of nonirradiated and UVA1-irradiated mice by immunohistochemical and RT-PCR analyses. Similar to our previous results [19], some infiltrating mononuclear cells expressed H2R in the LE-like skin lesions, and the expression of H2R mRNA was significantly up-regulated (data not shown). However, in nonlesional skins of MRL/lpr mice, there was no significant change in H2R mRNA expression between the nonirradiated and UVA1-irradiated mice.

 In addition, we analyzed the mRNA expressions of TNF-*α*, IFN-*γ*, IL-4, IL-10, and TGF-*β*. In the LE-like skin lesion, we could observe more remarkable mRNA expressions of TNF-*α*, IL-10, and TGF-*β* compared with nonlesional skin of nonirradiated mice, as reported in the previous study [20]. However, in the nonlesional skins, there were no significant changes between the nonirradiated and UVA1-irradiated mice (data not shown). Briefly, within our investigations, we could not detect any significant changes associated with the protective effects of UVA1 irradiation, regarding cytokines and HRs.

### 3.5. Induction of Apoptosis of Dermal Cells with UVA1 Irradiation

**Figure 6:**
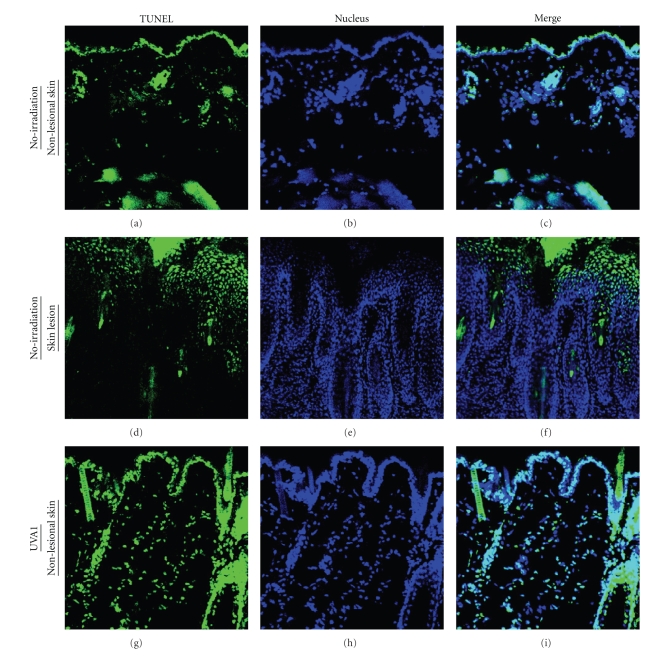
TUNEL assay for the analysis of apoptotic cells in the skin of MRL/lpr mice. (a), (b), (c): nonlesional skin of nonirradiated mice. (d), (e), (f): spontaneous LE-like skin lesion of nonirradiated mice. (g), (h), (i): nonlesional skin of UVA1-irradiated (10 J/cm^2^) mice. Positive cells for TUNEL assay are shown in green, whereas the nuclei are labeled with Hoechst 33342 in blue. In the nonlesional skin of the UVA1-irradiated (10 J/cm^2^) mice, TUNEL-positive cells were scattered in the dermis (g), (i). However, TUNEL-positive cells were rarely seen in the dermis of the nonlesional skin of the nonirradiated mice (a), (c). In the LE-like skin lesions, TUNEL-positive cells were diffusely detected in the hypertrophic epidermis, whereas in the dermis TUNEL-positive cells were hardly seen (d), (f). (a), (d), (g): TUNEL assay, (b), (e), (h): nuclear staining, (c), (f), (i): merged images of TUNEL assay and nuclear staining images, original magnification, ×200).

**Figure 7:**
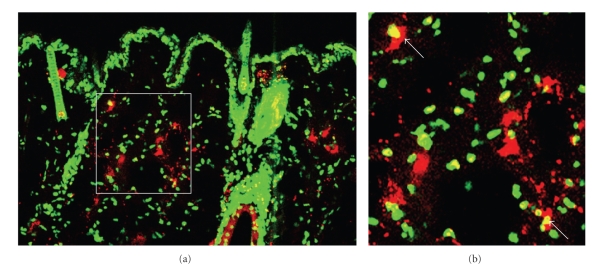
TUNEL-positive cells and mast cells in the UVA1-irradiated skin of MRL/lpr mice. Positive cells for TUNEL assay are shown in green, whereas mast cells are labeled with avidin-Texas Red to be shown in red, and merged images are shown. In the skin of UVA1-irradiated (10 J/cm^2^) mice, some double immunopositive apoptotic cells (yellow, indicated by arrows) were seen. (a): original magnification, ×200, (b): magnified image of the indicated region of (a).

The apoptosis of infiltrating cells has been recognized as one of the main mechanisms responsible for UVA1 treatment [16, 17, 28]. In the present study, TUNEL-positive cells were most remarkably seen in the dermis of the UVA1-irradiated mice among the three groups (Figure 6(i)). On the contrary, in the dermis of the nonirradiated mice without skin lesion, TUNEL-positive cells were scarcely detectable (Figure 6(c)). Although TUNEL-positive cells were seen diffusely in the epidermis of the nonirradiated mice with skin lesions, there were only a few TUNEL-positive cells in the dermis (Figure 6(f)). Next, we determined the types of cells showing TUNEL positive in the UVA1-irradiated skins with confocal microscopy. We could confirm that some of TUNEL-positive cells were mast cells (Figures 7(a), 7(b), indicated with arrows). The apoptosis of dermal mast cells might be closely associated with the reduction of the development of LE-like skin lesions in the UVA1-irradiated group. Thus, UVA1 irradiation might induce the apoptosis of pathogenic mast cells, eventually attenuating the development of spontaneous LE-like skin lesions of MRL/lpr mice.

Our study demonstrated that mast cells might play a role in the development of spontaneous LE-like skin lesions of MRL/lpr mice. Although we also speculated the possibility that UVA1 irradiation induced such mast cells as aggressively inhibited the development of LE-like skin lesions, for example, through the production of IL-10, we could not confirm such mast cells in the skin of UVA1-irradiated mice. Thus, this implied that UVA1 irradiation might induce the apoptosis of dermal pathogenic mast cells. In consistent with our results, apoptosis of proliferating mast cells was reported to be induced by UV in vitro, while resting skin mast cells were resistant to UV light-induced apoptosis [29]. However, as shown in Figure 7, UVA1 irradiation also induced the apoptosis of other types of dermal cells, except mast cells. Characterization of these apoptotic cells in the dermis of UVA1-irradiated mice still remained unclear, and we need further investigations, because several recent lines reported the new insights on autoimmunity in MRL/lpr mice through the impaired regulation of Langerhans cells [30] or regulatory T cells [31, 32]. Besides mast cells, these various immunocytes are speculated to be involved in the development of spontaneous LE-like skin lesions of MRL/lpr mice, and also the protective effects on them by UVA1 irradiation. In this context, the apoptosis of mast cells by UVA1 irradiation should be considered to contribute, but only in part, to the effects of UVA1.

In human LE, there have been only few reports investigating mast cells in the skin lesions [33]. Studies on 7 Japanese cases with chronic cutaneous LE revealed a 1.5-fold increase in infiltrating mast cells compared with the control skin [34]. Although evidence is lacking, mast cells are considered good candidates to mediate the tissue damage with autoantibodies and immune complexes in SLE [35].

## 4. Conclusions

In the present study, we demonstrated that UVA1 irradiation prevented the spontaneous development of macroscopic LE-like skin lesions of MRL/lpr mice. On the contrary, UVA1 irradiation did not have harmful systemic effects. As one of the mechanisms for the protective effects of UVA1 irradiation, we could suggest the induction of apoptosis of dermal pathogenic mast cells. These observations supported the possibility that UVA1 phototherapy would be one of the more rational treatments for skin lesions of SLE patients.

## Figures and Tables

**Table 1:** Effects of UVA1 irradiation on the development of spontaneous LE-like skin lesions in MRL/lpr mice.

Mice	Skin lesions			Total
	−	+	++	
Nonirradiated	12	4 (18.2)	6 (27.3)	22
UVA1	31	5 (13.9)	0	36_*#*_
5 J/cm^2^	16	2 (11.1)	0	18_$_
10 J/cm^2^	15	3 (16.7)	0	18

(++): mice with macroscopic skin lesions; (+): mice with only histological change in the skin; (−): mice without skin lesions; Regarding the frequency of spontaneous LE-like skin lesions in MRL/lpr mice, there was a significant difference between the non-irradiated and all UVA1-irradiated groups (*#*, *p* < 0.05), and between the non-irradiated and UVA1-irradiated (only 5 J/cm^2^) group ($, *p* < 0.05). UVA1 irradiation significantly protected mice against the development of LE-like skin lesions.

**Table 2:** *Mortality of MRL/lpr mice during experimental period.* Regarding the mortality of MRL/lpr mice during the experimental period, UVA1 irradiation tended to decrease the mortality of mice, but not significantly.

Mice	Total number	Number of dead mice (%)
Nonirradiated	22	5 (22.7)
UVA1	36	7 (19.4)
5 J/cm^2^	18	4 (22.2)
10 J/cm^2^	18	3 (16.7)

There were no significant differences among three groups.
